# Common measures or common metrics? the value of IRT-based common metrics

**DOI:** 10.1186/s41687-023-00657-w

**Published:** 2023-11-20

**Authors:** Caroline B. Terwee

**Affiliations:** 1 Department of Epidemiology and Data Science, Amsterdam UMC, Vrije Universiteit Amsterdam, Amsterdam, the Netherlands; 2 Amsterdam Public Health Research Institute, Methodology, Amsterdam, the Netherlands

**Keywords:** Patient-reported outcomes, Linking, Metric

## Abstract

There is a clear need to harmonize outcome measurement. Some authors propose to express scores as T scores to facilitate interpretation of PROM results in clinical practice. While this is a step in the right direction, there are important limitations to the acceptance of the T score metric as a common metric when T scores are based on raw sum scores of ordinal items: Such T scores of different instruments are not exactly comparable because they are not interval scaled; T scores of different measures are only on the same scale if exactly the same reference group is used; and the T sore metric cannot be maintained because it is reference population-dependent and needs to be updated regularly. These limitations can be overcome by using an item response theory (IRT)-based metric. Items from different measures can be placed on the same IRT metric to make scores comparable on an interval scale. The PROMIS initiative used IRT to develop item banks for measuring various health outcomes. Other PROMs have been linked to the PROMIS metric. Although PROMIS uses a T-score metric for practical reasons, the underlying PROMIS metric is actually an IRT metric. An IRT approach also enables further development of an item bank while preserving the underlying metric. Therefore, IRT-based metrics should be considered as common metrics for the future.

## Introduction

There is an urgent need to harmonize outcome measurement. Despite international efforts to harmonize outcomes measured in clinical trials and clinical practice (by using core outcomes sets (COS) [1]) or standard sets [2]), still many different outcomes are being measured and different outcome measurement instruments are still being used for measuring the same outcome. The uptake of COS is still low in most research areas [3]. Many challenges in implementing standard sets have been identified by the International Consortium for Health Outcomes Measurement (ICHOM), including cost-related issues for the use of proprietary or licensed patient-reported outcome measures (PROMs) [4]. Many of these issues have been highlighted by researchers who propose the implementation generic sets of PROMs instead of condition-specific sets of PROMs [5]. Nevertheless, disease-specific PROMs are still being developed and published almost daily.

One step forward are efforts to make scores of different instruments measuring the same outcome comparable. The use of different measures with different scales complicates the interpretation of test results and hampers the implementation of outcome measurement in healthcare. The psychometric process used to establish a relationship between the scores of two (or more) instruments is generically referred to as linking [6]. Linking procedures have been applied in the educational field for decades and are increasingly used to link patient-reported outcomes (PROs). Initiatives such as PROsetta Stone® (www.prosettastone.org ) and Common Metrics (www.common-metrics.org) developed and applied linking methods to link instruments such as Patient-Reported Outcomes Measurement Information System (PROMIS®) instruments with related instruments to provides equivalent scores for different scales that measure the same health outcome [7].

Different linking methods and different common metrics (the scales on which the scores are expressed) have been proposed in the literature [6, 8]. Some methods are based on simple statistics, such as means and variances or percentile ranks, while others use more complex models, such as item response theory (IRT) models, which place more demands on the data. Different methods have different advantages and disadvantages.

In the clinical psychological literature it has often been suggested to use standardized metrics such as z-scores or T-scores to facilitate interpretation of PROM scores [9, 10]. Recently, de Beurs et al. promoted this approach to facilitate interpretation of PROM results in clinical practice [8]. T scores are Z-scores (i.e. scores converted to a standard scale with a mean of 0 and SD of 1 based on the mean and SD of a reference group) multiplied by 10 with 50 points added. T scores are often used to facilitate interpretability of scores, for example in PROMIS instruments. T scores are attractive because they have a simple interpretation: A T score of 50 then represents the average score of the reference population, a score of 40 or 60 represents a score that is one SD lower or higher than the reference population, respectively. For example, a score of 60 on a PROMIS Depression measure indicates that the individual scores one SD higher (indicating more depressive symptoms) than the average general population [11].

While the proposal to express scores as T scores is a step in the right direction to standardize outcomes, there are important limitations to the acceptance of the T score metric as a common metric when T scores are based on raw sum scores. These limitations can be overcome by using an item response theory (IRT)-based metric. It should be noted that PROMIS T scores are not transformed raw sum scores but transformed IRT-based scores. For practical reasons the PROMIS initiative decided to convert the IRT metric to a T score metric to avoid negative scores and facilitate interpretation of the scale by shifting the metric in such a way that a T score of 50 represented the average of the US population with a SD of 10. However, the benefits of the underlying IRT-based theta metric were retained. The aim of this paper is to address the limitations of the T score metric as a transformation of raw sum scores and highlight the value of IRT-based metrics as common metrics.

## What is a common metric?

A common metric is an agreed unit or scale of measurement that is applied to data from different sources measuring the same construct. A well-known common metric is the Celsius scale (or Fahrenheit) for measuring temperature. We use different kind of thermometers to measure the temperature of our body when we are ill, the temperature in the oven when we bake a cake, and the temperature outside the house when we go for a hike. These thermometers also measure in different ranges of the temperature scale, e.g. the body thermometer measures in the range of 35–42 degrees Celsius, while the thermometer for the oven measures in the range of 100–250 degrees Celsius. Despite these different thermometers and different measurement ranges, the underlying measurement scale is the same Celsius scale. These thermometers measure the construct temperature on a common metric.

This is not the case for most clinical tests and patient-reported outcome measures (PROMs). For example, different PROMs exists to measure depressive symptoms, such as the Center for Epidemiologic Studies Depression scale (CES-D), Beck Depression Inventory-II (BDI), and the 9-item Patient Health Questionnaire (PHQ-9). The scale of the CES-D ranges from 0 to 60, the BDI scale ranges from 0 to 63, and the PHQ-9 scale ranges from 0 to 27. These scales are not the same and scores are not comparable; there is no underlying common depression metric.

Different solutions have been proposed to solve this problem. De Beurs et al. present an overview of some possible solutions and argue that the T score metric is the best choice [8]. An advantage of the T score metric is that it can be interpreted universally, provided a general population reference group is used. A T score of 50 then represents the average score of the general population, a score of 40 or 60 represents a score that is one SD lower or higher than the general population, respectively.

## Limitations of the T score metric as a common metric

There are four limitations to the acceptance of the T score metric as a common metric when based on raw sum scores. First, T scores of different instruments are not exactly comparable when T scores are based on raw sum scores because T scores are not interval scaled if the underlying scales of the tests are ordinal. Transforming an ordinal scale into a Z-score or T score, does not make it an interval scale. This means, for example, that the distance between a T score of 45 and 50 may not be the same as the distance between a T score of 40 and 45 and a the distance between a T score of 45 and 46 is not necessarily the same for the BDI’s T score scale as for the PHQ-9’s T score scale.

Second, T scores of different measures are only on the same scale if exactly the same reference group is used. If different general population samples are used for obtaining T scores of the BDI and the PHQ-9 (e.g. the population samples were obtained in different years or in different countries), the scores will not be on the same scale because the means and SDs of the samples may differ (see for an example Terwee et al. [12]). For example, a T score of 40 on the BDI represents a score that is one SD lower than the general population sample that was used for defining the T score metric of the BDI. However, it may represent a score that is e.g. 0.9 SD lower or 0.1 SD higher than the general population sample that was used for defining the T score metric of the PHQ-9. Using different reference groups is confusing and lead to incomparable scores. To obtain a really similar T score depression metric, one would have to administer all existing depression measures to the same sample, which is practically not feasible.

Third, the T sore metric cannot be maintained because it is reference population-dependent. When a new depression measure is developed, a T score metric for this new instrument comparable to the T score metrics of the BDI and PHQ-9 can only be obtained by recruiting a new general population sample to complete all depression questionnaires. However, the original T score metrics of the BDI and PHQ-9 will then not remain. This makes the T score metric not a sustainable solution.

Fourth, the T score metric needs to be updated regularly to ensure that a T score of 50 still represents the average score of the general population, since the health of general populations may change over time.

## The value of an IRT-based common metric

A solution to the above mentioned limitations is to use IRT linking methods [6, 13]. In an IRT model, all items of a measure that measure the same construct are calibrated on an underlying theta metric, with a mean of 0 and SD of 1 in the calibration sample. The theta metric is a real interval scale. Each item (or its response options in more complex models) has a unique location on the theta metric. Items from different measures can be placed on the same theta metric, for example through simultaneous calibration of items from multiple measures, or by fixing the item parameters of one measure and calibrate the item parameters of items from other measures on the fixed metric. If the item parameters of the co-calibrated or fixed IRT model are then used to estimate person scores, the scores are on the same (interval-scaled) metric [6, 13].

The PROMIS initiative used IRT to develop, among others, the PROMIS Depression item bank with an underlying PROMIS Depression theta metric [11]. Again, the conversion of IRT-based scores to T scores was done for practical reasons only. Once IRT scores are established, the final form of the scores are secondary to the construction. Other PROMs have been linked to the PROMIS depression metric using a linking approach as described above. For example, Choi et al. converted scores of the CES-D, BDI, and PHQ-9 to the PROMIS Depression metric [14]. With the IRT-based approach it is not necessary for all depression instruments to be administered to the same sample, as long as the samples receive a few PROMIS items along with the PROM to be linked.

An IRT-based common metric is also sustainable because the metric can be fixed. The IRT approach enables further development of an item bank while preserving the underlying metric. Items can be removed from, or added to an item bank and even the item locations of individual items can be shifted based on new evidence, but the underlying metric will remain the same^1^. The Celsius scale for temperature was established in 1742 and has not been changed since. The PROMIS initiative aims to maintain the PROMIS metrics by using the original calibrations of the item banks as the fixed PROMIS metrics.

A limitation of the IRT approach is that several assumptions must be met to apply IRT and linking, such as unidimensionality and a high correlation between the PROMs that are to be linked [6]. Also, large sample sizes are required to obtain reliable results.

Another limitation of an IRT-based metric is that, as with the T score metric, the interpretation of the metric may vary across groups and may change over time. While the average PROMIS Depression score of the US general population was 50 in the year 2000, the current US population or the Dutch population may score differently. So the value of 50 may not have the same meaning in all populations. This limitation could perhaps be overcome by simply stating that 50 is the “middle of the scale”, and population reference values can vary across the scale.

## Conclusion

There is a clear need to harmonize outcome measurement. Linking scores of different PROMs measuring the same construct on the same scale is one step in the right direction. Expressing scores as T scores is a relatively simple solution, but there are important limitations to the acceptance of the T score metric as a common metric when T scores are based on raw sum scores. These limitations can be overcome by using an item response theory (IRT)-based metric.

## Data Availability

Not applicable.

## Footnotes

1 In some cases, it was necessary to develop a new metric for a PROMIS domain based on new evidence, leading to a new version of the item bank.
